# Nanoparticle-Imprinted Silica Gel for the Size-Selective Capture of Silver Ultrafine Nanoparticles from Water

**DOI:** 10.3390/molecules28104026

**Published:** 2023-05-11

**Authors:** Piersandro Pallavicini, Luca Preti, Maria L. Protopapa, Daniela Carbone, Laura Capodieci, Yuri A. Diaz Fernandez, Chiara Milanese, Angelo Taglietti, Lavinia Doveri

**Affiliations:** 1 Department of Chemistry, University of Pavia, V.le Taramelli 12, 27100 Pavia, Italy; 2 ENEA-Italian National Agency for New Technologies, Energy and the Sustainable Economic Development, Division Sustainable Materials-Brindisi Research Center, S.S. 7 Appia km. 706, 72100 Brindisi, Italy

**Keywords:** silica gel, silver nanoparticles, gold nanoparticles, nanoparticles uptake, nanoparticles disposal, size selectivity

## Abstract

A synthetic approach has been developed to prepare silica gel monoliths that embed well separated silver or gold spherical nanoparticles (NP), with diameters of 8, 18 and 115 nm. Fe^3+^, O_2_/cysteine and HNO_3_ were all successfully used to oxidize and remove silver NP from silica, while aqua regia was necessary for gold NP. In all cases, NP-imprinted silica gel materials were obtained, with spherical voids of the same dimensions of the dissolved particles. By grinding the monoliths, we prepared NP-imprinted silica powders that were able to efficiently reuptake silver ultrafine NP (Ag-ufNP, d = 8 nm) from aqueous solutions. Moreover, the NP-imprinted silica powders showed a remarkable size selectivity, based on the best match between NP radius and the curvature radius of the cavities, driven by the optimization of attractive Van der Waals forces between SiO_2_ and NP. Ag-ufNP are increasingly used in products, goods, medical devices, disinfectants, and their consequent diffusion in the environment is of rising concern. Although limited here to a proof-of-concept level, the materials and methods described in this paper may be an efficient solution for capturing Ag-ufNP from environmental waters and to safely dispose them.

## 1. Introduction

Particles with a size lower than 100 nm are defined as ultrafine nanoparticles (ufNP). There is a great concern on the environmental and health effects of ufNP, especially of metal ones [1,2], due to the toxicity of many metals and to the increased oxidizability intrinsic to nano dimensions. The origin of metal ufNP may be unintentional, e.g., due to waste incinerators or traffic [1]. However, metal ufNP are also produced on purpose along the enthusiastic burst of nanotechnology over the past 30 years. They are incorporated in products and goods such as textiles [3], paints [4], coatings [5], fuel additives [6], plastics [3]. The use and disposal of such goods may release metal ufNP to air, water, soils [7]. Particular concern is raised by silver ufNP (Ag-ufNP) [8]. These have received enormous attention due to their large-spectrum antimicrobial action, with silver NP being used in fabrics, medical devices, disinfectants, household appliances, furniture, toothbrushes, clothes [9,10,11]. It has been estimated that more than 500 tons of Ag nanoparticles are fabricated per year [12]. We have been working for more than fifteen years on the synthesis of Ag-ufNP and on their use in antimicrobial materials and devices [13,14,15,16]. In this area, spherical Ag-ufNP with diameter 5–10 nm are typically employed, obtained from the popular and straightforward AgNO_3_/citrate/borohydride synthetic protocol in water [13,15,17]. We have now also begun to be concerned about the disposal of Ag-ufNP with such tiny dimensions. In this regard, we have recently found that Ag-ufNP with d = 8 nm are dissolved (i.e., oxidized) by O_2_ in water within a few hours if cysteamine (HS(CH_2_)_2_NH_2_) is added, thanks to the Ag^+^ coordinating ability of this molecule [18]. On the contrary, if Ag^+^ ligands are not used to promote Ag oxidation, Ag-ufNP may remain intact for a much longer time, also due to the low O_2_ solubility in pure water (8.85 mg/L at 20 °C [19]). In agreement with this, it has been shown that, in natural water environments, it takes several days or even weeks before Ag-ufNP are fully oxidized to Ag^+^ [20]. It is thus of obvious importance to have tools capable to remove Ag-ufNP from water, and that possibly also allow their straightforward disposal, i.e., transformation into non-nano materials.

**Scheme 1:**
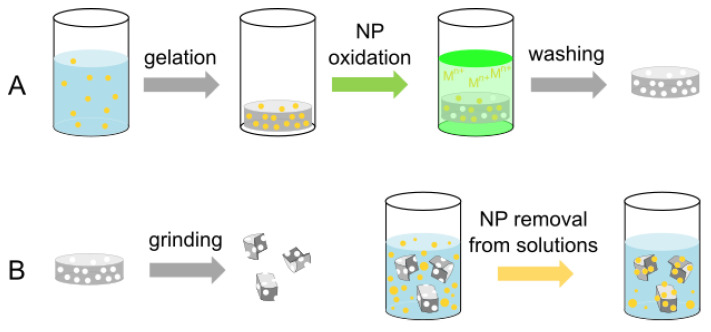
Working scheme for the preparation of NP-imprinted silica gel monoliths (**A**) and their use for the selective reuptake of NP of the same size from an aqueous colloidal solution (**B**).

Following our experience with molecularly imprinted polymers/NP hybrids [21,22] and the incorporation of Ag-ufNP in polymeric matrices [23], we show here that under proper conditions (reagents, solvent, pH, coating), Ag-ufNP resist intact in a solution undergoing the formation of silica gel. As sketched in Scheme 1A, a solubilized ionic or molecular oxidant can diffuse in a solid silica monolith, reach the embedded Ag-ufNP, and dissolve them via oxidation, leaving holes with the same shape and dimensions of the nanoparticles. We also show that the surfaces of the mechanically grinded emptied silica monoliths are able to re-uptake Ag-ufNP from an aqueous solution, Scheme 1B, exerting size selectivity based on the good match between the dimensions of the holes and those of the NP. A related approach has been described in the literature only by Mandler and coworkers [24]. With the Langmuir–Blodgett technique, they transferred onto an indium-tin oxide (ITO) support a polyaniline layer with embedded gold ufNP (Au-ufNP, d = 15 and 33 nm). NP were then removed via electro-oxidation and the cavities left behind were able of reuptaking AuNP with size-exclusion properties. The same authors later developed this elegant approach towards size-selective detecting systems for Au-ufNP [25,26]. In the present paper, we describe a convenient protocol to obtain bulk silica monoliths with embedded spherical Ag-ufNP with diameter 8 nm, or Au-ufNP with 18 nm diameter, or larger AuNP with 115 nm diameter. We then studied the wet chemical dissolution of the NP embedded in the monoliths by oxidation with O_2_/cysteamine, Fe^3+^, HNO_3_ (for Ag-ufNP) or aqua regia (for Au-ufNP and AuNP). Finally, we demonstrated the efficient and size-selective reuptake of the Ag-ufNP with d = 8 nm from water, using the grinded silica gel with corresponding 8, 18 or 115 nm void sites. The following procedures were used: transmission and scanning electron microscopy (TEM and SEM, respectively) performed before and after NP removal support our conclusions, together with full chemical-physical characterization, including porosimetric analysis, FT-IR spectra, X-ray powder diffraction, thermogravimetric analysis (TGA), absorption spectra and analytical determination of Ag and Au release. A model based on van der Waals attractive interactions is proposed to explain the observed size-selective reuptake by imprinted void silica materials.

## 2. Results and Discussion

### 2.1. Silica Monoliths with Embedded 8 nm Ag-ufNP

**Figure 1:**
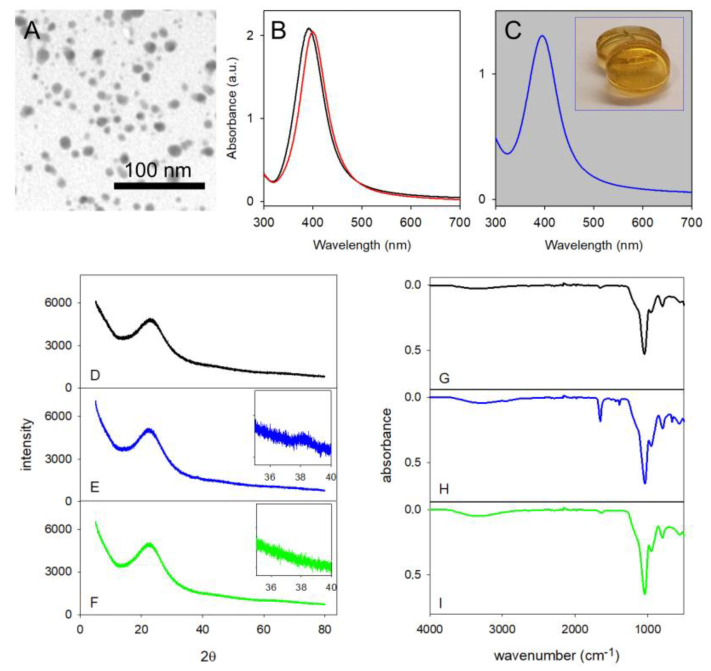
(**A**) TEM image of the citrate-coated 8 nm Ag-ufNP; (**B**) absorption spectra of colloidal solutions of citrate-coated 8 nm Ag-ufNP (black) and of the same after pegilation (red); (**C**) absorption spectra obtained on **Ag@SiO_2_-8**, i.e., the monolith with embedded 8 nm Ag-ufNP (inset: photography of two monoliths); (**D**–**F**) XRD diffraction on grinded monoliths (**D**) SiO_2_; (**E**) **Ag@SiO_2_-8**, inset = zoom on Ag (111) plane reflection; (**F**) void **SiO_2_-8** after Ag-ufNP removal with Fe^3+^, inset = zoom on Ag (111) plane reflection (not detected); (**G**–**I**) FTIR spectra ((**G**) SiO_2_; (**H**) **Ag@SiO_2_-8**; (**I**) void **SiO_2_-8** after Ag-ufNP removal with Fe^3+^).

Silica monoliths were first prepared containing spherical Ag-ufNP with d = 8 nm. The latter are a recurring type of AgNP used for biomedical applications, and our primary goal was to have imprinted void silica materials capable of reuptaking such particles. We prepared aqueous colloidal solutions of citrate-coated Ag-ufNP with d = 8 (±4) nm using a standard protocol [13] (TEM image in Figure 1A). In these solutions the total Ag is 5.78 × 10^−4^ M. We the grafted HS-PEG_750_ (polyethylene glycol thiol, average mw 750) on Ag-ufNP, obtaining pegylated Ag-ufNP (PEG_750_ is 48.8% *w*/*w* on AgNP, as calculated by TGA see Figure S1. While citrate-coated Ag-yfNP quickly aggregated, the pegylated Ag-ufNP were stable in the silica-gel precursor mixture (Figure S2). We used a base-catalyzed gelation protocol [27], with 1:1:1 *v/v* PEG-coated Ag-ufNP solution, TMOS and methanol, with the addition of small volumes of DMF, that allowed to obtain homogenous monoliths with no fractures [28]. The formation of multiple identical solid disk monoliths was obtained by pouring 0.5 mL of the precursor mixture in the wells of a 24-multiwell plate (wells diameter = 1.7 cm). A solid product formed in 4.5 h. The gel monoliths were allowed to age for 6 days at RT. Then, they were heated for 1 day to remove residual solvent traces and excess water.

The dry gel disks (**Ag@SiO_2_-8**) were transparent, with the yellow-orange color typical of Ag-ufNP (Figure 1C, inset). The citrate-coated particles have λ_max_ at 392 nm in water, Figure 1B black spectrum. Coating with HS-PEG_750_ shifts λ_max_ to 399 nm, Figure 1B, red spectrum. Embedding the Ag-ufNP in a SiO_2_ matrix maintains the absorption peak almost unaltered (Figure 1C) with λ_max_ 395 nm. This indicates that the particles are well separated in the solid monoliths, as a close approach or contact between particles would promote the LSPR band hybridization with peak enlargement and appearance of new bands at longer wavelengths [18]. Particle separation is mandatory to obtain single and separate spherical void cavities after Ag-ufNP removal, which are needed for an optimum size-selective reuptake, as aggregates would instead lead to larger cavities with undefined shape. Colloidal solutions of the PEG-coated Ag-ufNP were also stable during tangential ultrafiltration. This technique allowed us to reduce the volume of an Ag-ufNP solution from 100 to 2.5 mL, increasing the total Ag concentration to 0.0164 mol/L. After purification with a centrifugation (30 min, 15,000 rpm) and redissolution cycle also this concentrated solution was used to prepare SiO_2_ monoliths (photographs in Figure S3). XRD diffraction run on the grinded monoliths (obtained from Ag-ufNP solutions with 5.78 × 10^−4^ M Ag) revealed a profile similar to that of a grinded reference monolith of SiO_2_, prepared with the same protocol, Figure 1D,E. Only a large and weak peak is observed at 2θ = 38.1°, which is due to reflections from the Ag (111) plane (Figure 1E, inset). The weak and large shape of the signals is consistent with the small diameter of the Ag-ufNP (8 nm), as expected using the Scherrer equation [29]. ATR FTIR spectrum was run on a grinded monolith, Figure 1H. It displays the same rich peak pattern of a grinded blank SiO_2_ monolith (Figure 1G) at wavenumbers < 1050 cm^−1^, with additional sharp peaks at 665, 1385, 1419, 1446 and 1654 cm^−1^, which are attributed to the grafted HS-PEG_750_ and residual DMF.

### 2.2. Silica Monoliths with Embedded 18 nm and 115 nm Au-ufNP

**Figure 2:**
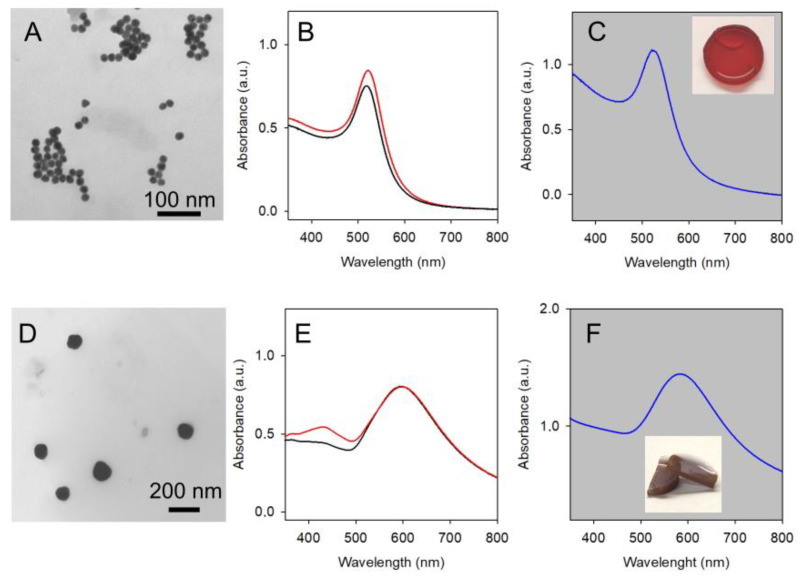
(**A**) TEM images of the 18 nm Au-ufNP; (**B**) absorption spectra of colloidal solutions of citrate-coated 18 nm Au-ufNP (black) and after pegilation (red); (**C**) absorption spectra obtained on the **Au@SiO_2_-18** monolith (inset: photography of a monolith); (**D**–**F**) same, for 115 nm AuNPs and **Au@SiO_2_-115** monolith.

In order to obtain silica with larger spherical cavities, we prepared silica monoliths with embedded Au-ufNP of 18 nm diameter. The choice of gold came from the difficulty of finding in the literature an easy and reproducible synthesis capable of producing silver nanospheres of ~20 nm diameter. We instead adopted the straightforward Turkevich approach [30,31] that gives reproducible access to citrate-coated Au-ufNP with d = 18 nm, Figure 2A. These have an LSPR absorption with λ_max_ 518 nm (Figure 2B) that shifts to 522 after coating with HS-PEG_2000_ with the method described in [32].

Assuming a ~100% transformation of HAuCl_4_ in Au (0) in the adopted synthesis (details in the experimental section), the total Au concentration in the colloidal Au-ufNP solutions is 2.4 × 10^−4^ M. The colloidal solutions of HS-PEG_2000_–coated Au-ufNP were also concentrated 100 times (total Au 2.4 × 10^−2^ M), via centrifugation (13,000 rpm) and redissolution of the pellets in 1/100 volume bi-distilled water. Monoliths containing 18 nm Au-ufNP particles (**Au@SiO_2_-18**) were prepared as for **Ag@SiO_2_-8**, using both as-synthesized and concentrated solutions. Absorption spectra on the monoliths containing the less concentrated Au-ufNP (photograph in Figure 2C, inset) display only a small shift of λ_max_ to 520 nm (Figure 2C), indicating also in this case that the NP embedded in the solid are well-separated.

Large AuNP with d = 115 nm were prepared with a modification of a described seed-growth synthesis [33] (details in the experimental section). Assuming a 100% conversion of Au(III), the total Au concentration in the product is 2.5 × 10^−4^ M. A TEM image is shown in Figure 2D. The absorption spectrum of Figure 2E (black line) reveals a maximum at 596 nm, with a weak shoulder at 430 nm. Pegilation followed, with HS-PEG_2000_ (see Experimental). The absorption spectrum maximum (Figure 2E, red line) does not change significantly on pegylation. Moreover, 100-fold concentrated solutions of pegylated 115 nm AuNP were obtained by centrifugating large volumes and redissolving the pellets in 1/100 volume of bi-distilled water. Silica monoliths containing large pegylated AuNP (**Au@SiO_2_-115**) were prepared both with standard and concentrated NP solutions, with the already described method. Figure 2F displays the absorption spectrum of the less concentrated **Au@SiO_2_-115** monolith, with λ_max_ at 580 nm. As in the case of **Au@SiO_2_-18** and **Ag@SiO_2_-8** the shape of the LSPR absorption peaks remains unaltered in the SiO_2_ gel monoliths, indicating well-separated, not aggregated NPs.

### 2.3. Void Imprinted Silica Gel

Void, NP-imprinted silica gels were obtained by oxidative processes, either as monoliths or as their grinded powders. In the case of **Ag@SiO_2_-8** the oxidants Fe^3+^, O_2_/HS(CH_2_)_2_NH_2_ and HNO_3_ were all successfully employed to prepare **void@SiO_2_-8**, according to Reactions (1)–(3),

Ag + Fe^3+^ ⇌ Ag^+^ + Fe^2+^ (1)

                    2Ag + ½O_2_ + 4HS(CH_2_)_2_NH_3_^+^ ⇌ 2[Ag(S(CH_2_)_2_NH_3_)_2_]^+^ + 2H^+^ + H_2_O (2)

         3Ag + NO_3_^−^ + 4H^+^ ⇌ 3Ag^+^ + NO + 2H_2_O (3)

which have been already described in the literature [18,34]. (Caution: NO is a toxic gas; therefore, Reaction (3) has to be carried out under a fume cupboard). The oxidation process can be followed by the eye with monoliths’ discoloration, when these are dipped in the oxidant solution. UV–Vis absorption spectroscopy allows one to follow the corresponding disappearance of the LSPR band of 8 nm Ag-ufNP at 390 nm, which was complete in 1 h and 10 h for O_2_/cysteamine and Fe^3+^ (Figure S4A and Figure S4B, respectively). A total of 30 s was instead sufficient when using HNO_3_ (concentrated, i.e., 63% *w*/*w* solution). When working on grinded monoliths powders, discoloration via oxidation was instead complete in a few seconds with all oxidants. In a control experiment, a **Ag@SiO_2_-8** monolith remained stable (no absorption spectrum changes) when dipped in bi-distilled water for 8 h (Figure S4C). XRD and FTIR experiments run on oxidized powders (Figure 1F,I) show profiles identical to those recorded on powders obtained from blank SiO_2_ monoliths, indicating the complete removal of both Ag-ufNP and of the grafted molecules. The total silver released from both monoliths and their grinded powders was analyzed via ICP–OES (inductively coupled plasma–optical emission spectroscopy), using 63% *w*/*w* HNO_3_ as oxidant and diluting the obtained solution to 2–5% *w/v* acid concentration. We found in both cases that >95% of the expected silver was released after 24 h contact with the oxidizing solution (the long time contact was chosen to ensure that all Ag^+^ was allowed to diffuse from the bulk silica to the receiving solution).

**Au@SiO_2_-18** and **Au@SiO_2_-115** were instead treated with aqua regia to obtain oxidation of Au nanoparticles to the corresponding SiO_2_ monoliths or powders with imprinted cavities, namely **void@SiO_2_-18** and **void@SiO_2_-115** (caution: aqua regia in an aggressive reagent that has to be prepared and used only under a fume cupboard). A fast process is observed on monoliths (discoloration time < 1 min), which becomes instantaneous when grinded powders are used. Additionally, the total gold released from both monoliths and their grinded powders was analyzed via ICP-OES after oxidation, finding the release to be >95% of the expected gold after 24 h.

XRD analysis carried out on grinded powders of **Au@SiO_2_-115** revealed peaks at 2θ values 38.1, 44.3, 64.5 and 77.7, relative to the standard (111), (200), (220) and (311) reflections, respectively, of FCC gold lattice (Figure S5). All reflections disappeared in **void@SiO_2_-115** grinded powders, obtaining a diffraction profile identical to that of grinded blank SiO_2_. FTIR analysis gave very similar results to those of **Ag@SiO_2_-8** and their void imprinted analogues, pointing towards the complete removal of both AuNP and their grafted molecules.

Porosity characterization was carried out via BET surface area analysis both on **Ag@SiO_2_-8** and **void@SiO_2_-8** monoliths and on their grinded powders, prepared with the standard Ag concentration (total Ag 5.78 × 10^−4^ M). The experimental nitrogen sorption isotherms (Figure S6) are in all cases typical of mesoporous materials [35,36]. Moreover, the type of hysteresis found for all the analyzed samples is of type H2(b), i.e., typical of materials presenting pores with a narrow pore body size W and a large neck size Wc (W > Wc) [36]. The specific surface area (SSA) contributed via micropores; the average pore radius values (calculated at P/P0 = 0.99 and by BJH analysis, see experimental for details) are listed in Table 1 for **Ag@SiO_2_-8** monoliths and grinded powders, and for the respective void materials **void@SiO_2_-8** (after Ag-ufNP oxidation by Fe^3+^).

From the data reported in Table 1, it is clear that the removal of 8 nm Ag-ufNP leads to an increase in SSA and in the area of the micropores compared to the starting situation, both in the case of monoliths and of their powders. However, the average pore radius remains small in all cases (<2 nm), indicating that the prevailing porosity is dictated by the intrinsically porous silica gel structure, to which the voids created by 8 nm Ag-ufNP removal superimpose with minor effects. From another point of view, it can be said that the number of pores created by the Ag-ufNP removal is much lower than the number of intrinsic, much smaller nanopores of the SiO_2_ gel structure. Comparable properties and a comparable behavior are observed when **Au@SiO_2_-115** monoliths and their void products, **void@SiO_2_-115** are examined (also in this case prepared with colloidal solutions in which the total Au concentration has the standard 2.5 × 10^−4^ M value). Table 1 reports an increase in the SSA and SSA contributed by micropores on AuNP removal, similarly to the case of **Ag@SiO_2_-8**. Moreover, also the average pore radius is small (<2 nm) and comparable to that of **Ag@SiO_2_-8,** and does not change significantly on AuNP removal. The prevailing porosity is dictated by the intrinsically porous gel structure and not by the presence or removal of nanoparticles. After observing the strict similarities of the results obtained with the smaller (8 nm) and larger (115 nm) nanoparticles, we opted to skip such experiments on the silica materials with Au-ufNP of intermediate dimensions (18 nm).

**Figure 3:**
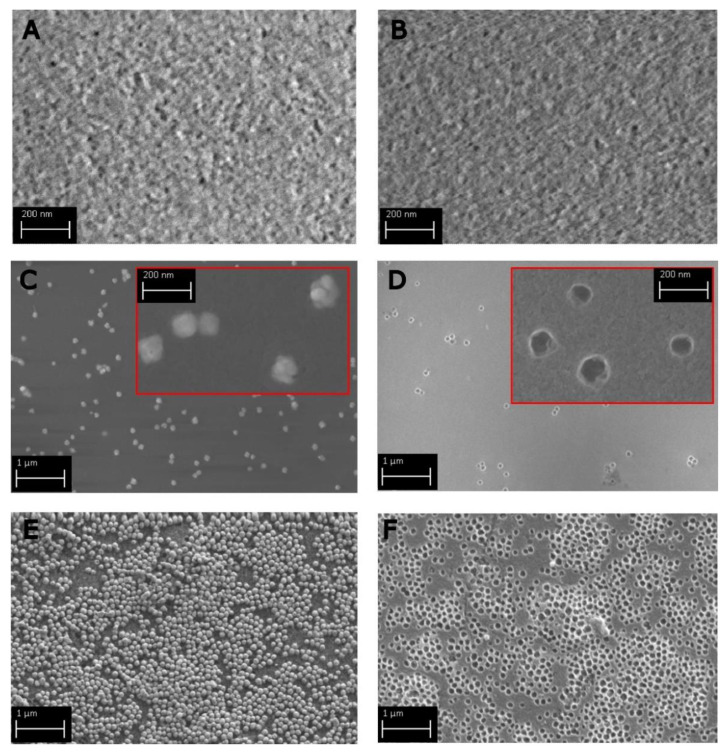
(**A**,**B**) SEM micrographs of a blank SiO_2_ monolith surface (**A**) and of the surface of a **Ag@SiO_2_-8** monolith (**B**). (**C**,**D**) same for an **Au@SiO_2_-115** monolith surface and its void counterpart at 20k× magnification (inset: detail at 100k× magnification). (**E**,**F**): same, repeated on an **Au@SiO_2_-115** monolith prepared with a 100× concentrated AuNP solution.

SEM imaging was carried out on the surface of blank SiO_2_ monoliths (Figure 3A) and compared with imaging of the surface of **Ag@SiO_2_-8** monoliths (Figure 3B). Even at high magnification (100k×), there are no significant differences between the two surfaces. The small dimensions of the Ag-ufNP and the conductive coating (~5 nm thick sputtered platinum layer) did not allow one to individuate the Ag-ufNP.

Expecting a similar picture when using 18 nm Au-ufNP, we skipped imaging on **Au@SiO_2_-18** and **void@SiO_2_-18**. SEM imaging was instead carried out on **Au@SiO_2_-115** monoliths and on their void counterparts, **void@SiO_2_-115**. In this case, the large AuNP are sharply evident on the monolith surface (Figure 3C). Once the AuNP are removed, the holes left behind have the expected corresponding dimensions, Figure 3D. To demonstrate that it is possible to emphasize the effect of added nanoparticles (i.e., to increase the number of holes in the materials), monoliths were also prepared using 100× concentrated colloidal solutions of pegylated 115 nm AuNP. Imaging on the obtained monoliths shows a dense layer of AuNP lying on the surface (Figure 3E), which leaves behind a comparable densely populated pattern of holes after oxidation with aqua regia (Figure 3F).

### 2.4. Uptake of 8 nm Ag-ufNP

**Figure 4:**
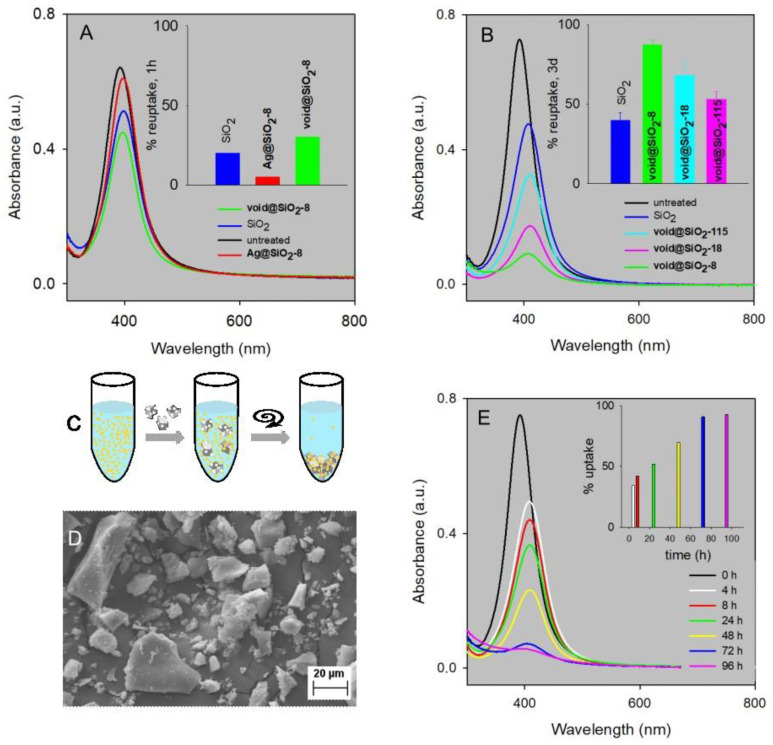
(**A**) absorption spectra of the supernatant from centrifugation after 1 h contact of **void@SiO_2_-8**, **Ag@SiO_2_-8** and plain SiO_2_ powders with a colloidal solution of Ag-ufNP (d = 8 nm, Ag = 5.8 × 10^−5^ M); the black spectrum is of the same colloidal solution, not treated with any silica powder, after centrifugation; inset: % of uptaken Ag-ufNP. (**B**) absorption spectra of the supernatant from centrifugation after 72 h contact of **void@SiO_2_-8**, **void@SiO_2_-18**, **void@SiO_2_-115** and plain SiO_2_ powders with a colloidal solution of Ag-ufNP (d = 8 nm, Ag = 5.8 × 10^−5^ M); the spectrum of the same colloidal solution, not treated with any silica powder, after centrifugation is also reported (black spectrum) for comparison; inset: % reuptake. (**C**) working scheme of the uptake process. (**D**) SEM image of grinded **void@SiO_2_-8**. (**E**) absorption spectra of the supernatant from centrifugation carried out after 0 h (i.e., no silica powder added), 4, 8, 24, 48, 72 and 96 h contact of **void@SiO_2_-8** with a colloidal solution of 8 nm Ag-ufNP (Ag 5.8 × 10^−5^ M); inset: % uptake vs. time.

Ag-ufNP uptake experiments were first carried out using powders obtained by grinding **void@SiO_2_-8** monoliths. The working scheme is sketched in Figure 4C.

In detail, powders were suspended in solutions containing 8 nm Ag-ufNP and kept for a given time under gentle shaking. Centrifugation allowed to separate the silica particles and the Ag-ufNP adhering to their surface from the supernatant solution, in which the not-uptaken Ag-ufNP remained. The concentration of the 8 nm Ag-ufNP left behind in the supernatant was measured via absorption spectroscopy. Comparison with the absorbance of a colloidal solution of 8 nm Ag-ufNP that underwent the same centrifugation treatment allowed to calculate by difference the % of uptaken NP (it has to be remarked that the absorbance of these control solutions decreased always less than 4% by centrifugation). Monoliths’ grinding was carried out manually with mortar and pestle. The fragments of the obtained powders have dimensions in the 1–50 µm range, as shown via SEM imaging (Figure 4D). Attempts to use powders prepared via ball milling failed, as the obtained fragments were so small that these were only partially separated via centrifugation, leading to turbid supernatants. In a typical experiment, 160 mg powder was suspended in 10 mL of 8 nm Ag-ufNP. We used an Ag-ufNP solution with total Ag concentration of 5.8 × 10^−5^ M. As a first experiment, we stirred for 1 h grinded powders of **void@SiO_2_-8**, and, for comparison, also of **Ag@SiO_2_-8** and of plain SiO_2_ monoliths. The results displayed in Figure 4A show that the highest uptake of Ag-ufNP is obtained with **void@SiO_2_-8** (30.1%). Its analogue with all-filled cavities, **Ag@SiO_2_-8** gives only 5% uptake. However, pure silica was able to uptake a significant 20.1% of 8 nm Ag-ufNP and, on this basis, we decided to always compare the uptake ability of grinded void gels with that of grinded pure silica. Investigation of the uptake kinetics was carried out by measuring the absorption spectra of the supernatant solution (containing the left-behind, not-uptaken 8 nm Ag-ufNP) at 4, 8, 24, 48, 72 and 96 h. The results (Figure 4E) show a linear increase of the % uptake with time, which reaches a plateau (>90%) after 3 days. On the basis of this result, 3 days was taken as the standard contact time in all the following experiments.

Increasingly concentrated 8 nm Ag-ufNP colloidal solutions were then experimented in uptake processes, ranging from total Ag 5.8 × 10^−5^ M to 5.8 × 10^−4^ M. 10 mL of the 8 nm Ag-ufNP solution and 160 mg of grinded **void@SiO_2_-8** were used for all concentrations. The % of uptaken Ag from solution is listed in Table 2.

The results of Figure 4 and Table 2 have been obtained with a **void@SiO_2_-8** material prepared via gelification of a precursor solution in which the total Ag concentration was increased using a concentrated solution of 8 nm Ag-ufNP (total Ag = 1.006 mg/mL, determined by ICP analysis). Monoliths were prepared as described, by adding 0.5 mL of this solution in each well, corresponding to 0.503 mg Ag per monolith (~80 mg dry monolith mass). As the standard mass of powder used for the reuptake experiments is 160 mg, before Ag oxidation, it contained 1.006 mg Ag. From the dimensions of a single particle (d = 8 nm) and Ag density, the average mass of a single 8 nm Ag-ufNP is calculated (2.811 × 10^−18^ g) and the average number of NP in 160 mg of powder is obtained (3.579 × 10^14^). After Ag-ufNP oxidation, this leaves behind the same number of cavities in 160 mg of **void@SiO_2_-8** powders. This is just a roughly calculated maximum number of imprinted cavities, and obviously not all the cavities are exposed even in grinded powders. However, it can be used for an evaluation of the % of occupied cavities in the **void@SiO_2_-8** powders after the reuptake process. Table 2 shows that on increasing the concentration of 8 nm uf-AgNP in the reuptake solution, the % of uptaken particles decreases, although remaining well over 50%. By converting in uptaken mass and number of uf-AgNP, we observe instead an almost linear increase with concentration. This sharply indicates that the **void@SiO_2_-8** powders have a high loading capacity that is not exhausted even at the highest examined Ag-ufNP(sol) concentration. An experiment carried out on an extremely highly concentrated Ag-ufNP solution (~1 × 10^−2^ M Ag) led to the uptake of 1080 μg Ag by a 160 mg sample of **void@SiO_2_-8** powder, with 107% of occupied cavities. This corresponds to 6.75 mg Ag/g powder. We take this as the limit reuptake capacity of the imprinted **void@SiO_2_-8** powder. As non-imprinted SiO_2_ powders also have a significant capacity to uptake 8 nm Ag-ufNP (see Figure 4A,B), we must consider that non-imprinted portions of the surface of **void@SiO_2_-8 silica** microparticles also contribute to NP reuptake, allowing a % of occupied cavities > 100. Finally, it has to be remarked that the mortar and pestle technique yields microparticles with a large dimensional and shape distribution (Figure 4D). Although we have not investigated this aspect, a more tuneable grinding technique that produces microparticles with a smaller average size would be expected to expose more cavities and further increase the uptake capacity of these materials.

### 2.5. Size Selectivity

**Figure 5:**
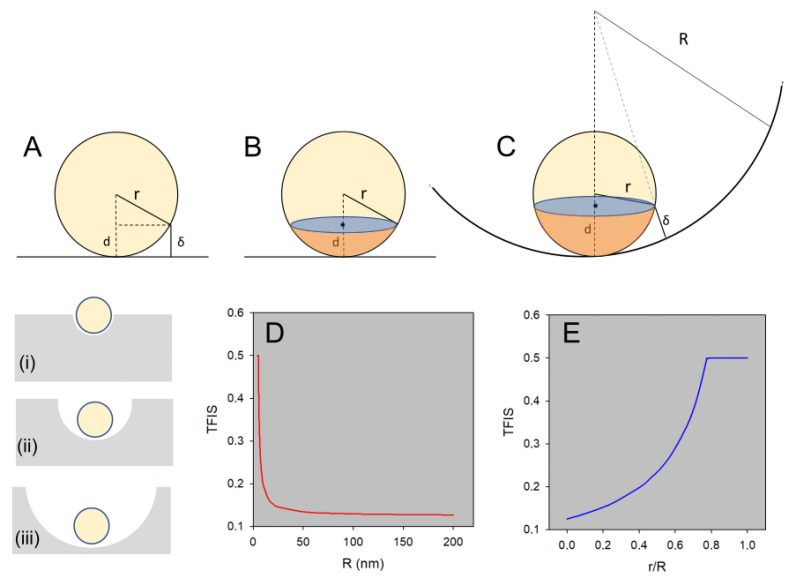
(**A**) sketch of a spherical NP with radius r in contact with a flat surface; δ is the minimum distance at which the Van der Waals attraction forces are significant; accordingly, only the NP portion at distance d ≤ δ contribute significantly to the Van der Waals attraction: this is the orange-colored portion of panel (**B**) When the same NP is inside a spherical cavity with curvature radius R panel (**C**) the NP portion interacting with the cavity surface increases (orange-colored portion). (**D**) theoretical fraction of interacting surface (TFIS) vs. cavity curvature radius) calculated for a NP with r = 4 nm using an exemplificative cut off distance δ =1 nm. (**E**) TFIS vs. r/R. Panel (**i**)–(**iii**) offers a visual sketch of the fit of a sphere of a given dimension (r = 4 nm) in a cavity with R = 4 nm (**ii**), 9 nm (**ii**) and >25 nm (**iii**).

The size selectivity of the reuptake process was also checked. Grinded powders of **void@SiO_2_-8**, **void@SiO_2_-18**, and **void@SiO_2_-115** were stirred for 3 days in a solution of 8 nm Ag-ufNP, at Ag concentration 5.8 × 10^−5^ M. All the powders were prepared so to have a comparable voids concentration as that of **void@SiO_2_-8**. The experiment was repeated 3 times, with grinded monoliths from different preparations. Figure 4B displays the series of spectra of the supernatant in one of the three repetitions. The inset reports the average % of uptake calculated over the 3 repetitions (standard deviation in parenthesis), that is 87(3)%, 68(7)% and 53(4)% for 8, 18 and 115 nm cavities, respectively. Low standard deviations indicate a good reproducibility of the materials and of their performances. The results sharply show that the void imprinted silica gels display reuptake selectivity, based on the best hole-sphere dimensional fit, as visually sketched in Figure 5, panel (i)–(iii). Moreover, all SiO_2_ gels with imprinted holes show a higher uptake of 8 nm Ag-ufNP than plain silica (40(4)%). In this regard, it is important to stress that the uptake experiments were carried out on citrate-coated Ag-ufNP in aqueous solutions with pH 5–6. In this pH range Ag-ufNP have a weak negative zeta-potential (−14 (±2) mV, at pH 5.5) while the surface charge of silica gel particles is also expected to be negative, as SiO_2_ has a point of zero charge (pzc) of ~2.0 [37].

Electrostatic interactions play thus an unfavourable role in the adhesion of Ag-ufNP on SiO_2_ powders. As a consequence, the observed uptake is to be attributed only to van der Waals (VdW) attractive forces. On this basis, a geometrical model allowed us to explain the observed hole-size selectivity. VdW interactions decay very fast with the distance (d) between the particle and the surface of a solid material. We can therefore define a cut off distance (δ), considering that only the fraction of the particle surface that is close enough to the solid surface (d ≤ δ) will contribute to the VdW attraction forces. Under this assumption, when a spherical particle interacts with a flat surface, see Figure 5A, only a small fraction of the sphere will contribute to the attractive forces, i.e., the section with orange color in Figure 5B. As an example, considering a cut off distance δ =1 nm and a spherical NP of d = 8 nm, it can be estimated that the fraction of the particle surface that interacts with a flat solid surface (TFIS, theoretical fraction of interacting surface) is only 0.12 (i.e., 12%, see Figure S7 for model details). This is visualized in Figure 5D for R →∞, where R is the curvature radius of a silica cavity that contains a NP of radius r. When the NP interacts with a curved surface with finite curvature, such as the inner surface of a cavity as in Figure 5C, maintaining the cut off distance δ constant the portion of interacting particle increases (orange colored portion). It is possible to calculate the fraction of the particle surface interacting with the curved cavity (see Figure S7), and in this case the TFIS will depend on the ratio between the particle radius and the radius of curvature of the cavity (R), Figure 5E, leading to a relative increase in the particle-cavity interactions as this ratio increases. It is important to note that in this model we have assumed that cavities are half-hemispheres, to allow for the NP to enter (i.e., when the size of the NP is close to the cavity radius, the NP can only enter the cavities that have been cut through at least by half). For this reason, the limiting value of the NP surface fraction is set at 0.5 in Figure 5D,E, when NP and cavity sizes become very similar, i.e., R − r ≤ δ. This assumption is not limiting the overall interpretation of the results and become irrelevant for cavities much larger than the NP size. This simple geometrical model can therefore explain the enhanced selectivity of our porous materials towards NPs, when the void-material cavity size matches the size of the adsorbed particles.

## 3. Materials and Methods

### 3.1. Materials

Silver nitrate ACS reagent, 99.0%; gold (III) chloride solution 99.9%; (30 wt % in dilute HCl); sodium borohydride ≥ 98.0%; L-ascorbic acid ≥ 99.0%; poly(ethylene glycol) methyl ether thiol (HS-PEG-OCH_3_, mw 2000 and mw 750); TritonX-100 (laboratory grade); tetrahydrofuran, ≥99.9%; potassium hexacyanoferrate (II), ≥98.5%; iron (III) chloride, 97%; iron(III) nitrate nonahydrate; hydroquinone, 99.0%; N,N-dimethylformamide, >99.0%; tetramethyl orthosilicate (TMOS), 98%; tetraethyl orthosilicate, ≥99.0%; sodium chloride, >99.5%; cetyltrimethylammonium bromide (CTAB); methanol, HPLC gradient grade, >99.9%; cysteamine hydrochloride, 97%; sodium hydroxide ≥ 98%, pellets; nitric acid ≥ 65%; hydrochloric acid ≥ 37% have all been bought from Merck Life Science (Milano, Italy), and used without further purification. Aqua regia is obtained by carefully mixing (under a fume cupboard) concentrated hydrochloric acid (37%) with concentrated nitric acid (63%) in 1:3 *v/v* ratio, and it is used immediately after preparation.

### 3.2. Methods and Instrumentation

All the glassware was treated before use with aqua regia for 15 min and then washed three times with bi-distilled water in an ultrasound bath for 5 min.

Centrifugation was carried out using an Hermle Z366 ultracentrifuge with polypropylene 10 mL tubes or 1.5 mL Eppendorf vials.

Ultrafiltration was carried out using the Hermle Z366 ultracentrifuge with a falcon rotor and centrifugal filters UltracelR-10 K. As an alternative we used a molecular weight cut off tangential filter (MWCO 5000) with a peristaltic pump.

Measurements of pH were carried out with a XS Instruments pH-meter (pH 50 model) with a Thermo Scientific Orion 91022 BNWP combined glass electrode. Electrode calibration was carried out before measurements, with buffer solutions at pH = 4, pH = 7 and pH = 10.

Absorption spectra were collected on a Varian Cary 60 Spectrophotometer, either in 1 mm or 1 cm optical glass cuvettes.

FT-IR Spectroscopy was performed with a FTIR Nicolet™ iS™ 50 instrument by Thermo Fisher Scientific, Milano, Italy, employing the ATR accessory equipped with a diamond crystal as internal reflection element (IRE). Spectra were recorded in absorbance ranging from 4000 to 400 cm^−1^ with 4 cm^−1^ in resolution.

Transmission Electron Microscopy (TEM): colloidal solutions from syntheses were diluted 10–100 times with bi-distilled water and 10 µL were dropped on nickel grids (300 mesh) covered with a Parlodion membrane, and then dried in a desiccator. Images were acquired by using a JEOL JEM-1200 EX II 140 microscope with a 100 kV acceleration voltage.

Scanning Electron Microscopy. Samples were sputtered with gold and observed with a ZEISS EVO MA10 microscope (Carl Zeiss, Oberkochen, Baden-Württemberg, Germany). Images were collected in high vacuum, at room temperature at different magnifications. It was also used a field-scanning electron microscope, provided with a Schottky ZEISS Meriln^®^ source and column GEMINI II, was used for the analysis of the samples structure and morphology.

Determination of Ag and Au by ICP-OES spectroscopy. An Ag or AuNP-loaded monolith or its grinded powders were kept dipped in the oxidant solution (5 mL) for 24 h (oxidant = 63% HNO_3_ or aqua regia, for Ag and Au, respectively). The receiving aqueous solutions were separated from the solid by decantation in the case of monoliths and by centrifugation in the case of powders. After dilution, the aqueous samples were analyzed for the metal content by ICP-OES with Perkin Elmer Optima 3300 DV instrument.

Porosimetric analysis. BET surface area analysis was performed by means of the nitrogen surface absorption technique (at the cryogenic temperature of 77 °K) using the AUTOSORB IQ system of the Quantachrome. To eliminate any traces of contaminants absorbed during the exposure to the atmosphere, the samples were initially pre-treated in an oven at a temperature of 60 °C for 24 h. Subsequently, the various samples were inserted inside the measuring cell for a degassing treatment in vacuum (about 0.1 Torr) at a temperature of 120 °C for 180 min with a heating ramp of 10 °C/min. The choice of degassing parameters was carried out to obtain an effective removal of moisture (or any volatile substances) from the surface of the sample without inducing any irreversible change.

X-ray diffraction analysis of the powders was carried out by means of a Multipurpose Empyrean (Malvern-Analytical) diffractometer equipped with Cu anode (Kα = 1.540 Å). The equatorial divergence of the incident X-ray beam was controlled using a Programmable Divergence Slit (Fixed 0.5°) with antiscatter slit (1°) and with Soller slit (0.04 rad.), while a Soller slit (0.04 rad.) with antiscatter slit (7.5 mm) was placed in front of the detector. The samples were placed on a sample stage for powders in spinning active mode (rotation time 16 s). A 2D solid-state hybrid pixel (PIXcel3D) was used as detector.

### 3.3. Syntheses

#### 3.3.1. Ag-ufNP, d = 8 nm

Silver nanoparticles of 8 nm diameter and coated with citrate were prepared using a published method [13]. Briefly, a 1% *w*/*v* AgNO_3_ solution, a 1% *w*/*v* sodium citrate solution, a 0.075% NaBH_4_ in 1% *w*/*v* sodium citrate and 100 mL of bi-distilled water are separately cooled in an ice bath for at least 20 min. Subsequently, the following additions are made to the 100 mL volume of water under vigorous stirring: 1 mL of AgNO_3_ solution, 1 mL of citrate solution and after a minute 0.5 mL of the NaBH_4_ solution. Stirring is stopped and the preparation is allowed to return to room temperature. A deep yellow-orange color is obtained after a few minutes.

#### 3.3.2. Au-ufNP, d = 18 nm

Gold nanoparticles of 18 nm diameter and coated with citrate were synthesized with the Turkevich method [30,31], using these quantites: 87 µL of a tetrachloroauric acid aqueous solution (1.44 M) are added to 500 mL of boiling bi-distilled water. The heating is switched off and, under magnetic stirring, 25 mL of a 0.017 M sodium citrate solution are also added. The solution turns deep red in a few minutes and it is kept under stirring for further 2.5 h.

#### 3.3.3. AuNP, d = 115 nm

Citrate-coated AuNP with average size 115 nm were synthesized by a seed growth method with slight modifications to a published method [33]. Spherical citrate-coated Au-ufNP with d = 18 nm were prepared as described in Section 3.3.2 using these quantities: 183 μL HAuCl_4_ solution (0.041 M), 30 mL bi-distilled water, 0.90 mL 0.034 M sodium citrate. This was the seeds solution, which was used within 1 day for the next step (growth). 610 μL of aqueous HAuCl_4_ (0.041 M) were added to 96 mL of bi-distilled water under vigorous stirring. In sequence, 500 μL of seeds solution, 220 μL of 0.034 M sodium citrate, and 1 mL of a 0.030 M hydroquinone in water were added in sequence. The solution was kept under stirring at room temperature for 30 min, after which time a dark brown-grey solution was obtained.

#### 3.3.4. Coating Nanoparticles with PEG Thiols

Citrate was displaced from silver and gold nanoparticles by coating them with poly(ethylene glycol) methyl ether thiols (HS-PEG), using HS-PEG with mw 750 for Ag-ufNP (8 nm) and with mw 2000 for Au-ufNP (18 nm) and AuNP (115 nm). In a typical preparation, solid PEG-SH of the chosen mw was added to 100 mL of the nanoparticles solutions (prepared as in Section 3.3.1, Section 3.3.2, or Section 3.3.3) so to have a 10^−5^ M concentration. The solution was allowed to react for 24 h under stirring at room temperature in a stoppered flask. For Au-ufNP (18 nm) and AuNP (115 nm) purification from excess PEG-SH was carried out by centrifugation (13,000 rpm), decantation of the supernatant, redissolution of the pellet in the starting volume of bi-distilled water (repeated for two cycles). For the smaller Ag-ufNP ultrafiltration was used as an alternative, either with centrifugal filters or with tangential filters with MWCO = 5000 connected to a peristaltic pump. After ultrafiltration, the retained volume was reintegrated to the starting volume with bi-distilled water. By this, solutions were prepared containing NP at the standard concentration, namely Ag 5.78 × 10^−4^ M (Ag-ufNP 8 nm), Au 2.4 × 10^−4^ M (Au-ufNP 18 nm), and Au 2.5 × 10^−4^ M (AuNP 115 nm). However, both processes allowed also on demand concentration. As an example, after centrifugation the obtained pellet can be redissolved in 1/10 volume vs. the starting one, obtaining a 10× concentrated solution. In a typical ultrafiltration purification process, a volume of 100 mL of pegylated nanoparticles is concentrated to 25 mL (4× concentration). Further ultrafiltration on gathered concentrated samples lead to the desired increased concentration. It has to be noted that the concentration processes may lead to some loss of the nanoparticles. In all cases, we checked the real final concentration by oxidizing a 0.5 mL sample of the solution (with HNO_3_ for Ag and aqua regia for Au) and determine the metal content by ICP-OES.

#### 3.3.5. Preparation of NP-Containing Silica Gel Monoliths (**Ag@SiO_2_-8**, **Au@SiO_2_-18**, **Au@SiO_2_-115**)

A total of 2.5 mL of the chosen HS-PEG coated Ag or AuNP solution (either at the standard concentration or at increased concentrations), 2.5 mL TMOS (Tetramethyl orthosilicate), 2.5 mL methanol, and 1.0 mL DMF are added in sequence in a 50 mL beaker and gently magnetically stirred. Then, 100 μL of 0.050 M aqueous NaOH is added. After stirring for 5 min, the wells of a 24-well plate were filled with 0.5 mL each of the solution. The plate was then covered with parafilm, and left to gel at room temperature for 3 days. Then, pinholes were made on parafilm and three days were spent for ageing. Finally, the multiwell plate with the formed monoliths was put in an oven at 70 °C for 24 h to eliminate the solvent residues.

#### 3.3.6. Preparation of Silica Gel Monoliths without NP

Plain silica monoliths were prepared as reference materials for all the chemical and chemical–physical characterizations carried out in this paper. The synthesis is identical to what described in Section 3.3.5, but 2.5 mL bi-distilled water was used instead of an NP solution. The obtained SiO_2_ monoliths were colorless, transparent, and crack-free.

#### 3.3.7. Removal of Ag-ufNP (8 nm), Au-ufNP (18 nm) and AuNP (115 nm) from Silica Gel Monoliths

The removal of Ag-ufNP allowed us to obtain **void@SiO_2_-8**, **void@SiO_2_-18** and **void@SiO_2_-115** monoliths. This was obtained via slow immersion of the chosen monolith in 5 mL of the oxidizing solution. The latter was either 0.1 M cysteamine hydrochloride, or 0.1 M Fe(III) nitrate, or HNO_3_ 63% for **Ag@SiO_2_-8** monoliths. Complete monoliths discoloration was observed within 5 min using HNO_3_, and within 1 day using either Fe(III) or O_2_/cysteamine. The removal of Au-ufNP and AuNP from **Au@SiO_2_-18** and **Au@SiO_2_-115** monoliths was carried using freshly prepared aqua regia, observing complete discoloration within 3 min. In all cases, the monoliths were kept in the oxidizing solution for 1 h after complete discoloration, then rinsed three times with water and kept in bi-distilled water for further 24 h. Before any further characterization or grinding, they were allowed to dry in an oven at 70 °C for 16 h.

#### 3.3.8. Preparation of **void@SiO_2_-8**, **void@SiO_2_-18** and **void@SiO_2_-115** Powders

Powders were easily obtained from **Ag@SiO_2_-8**, **Au@SiO_2_-18**, **Au@SiO_2_-115** monoliths by grinding with a mortar and pestle. The powder from one monolith (80 mg) or two monoliths (160 mg) was suspended in 5 mL HNO_3_ (**Ag@SiO_2_-8**) or aqua regia (**Au@SiO_2_-18**, **Au@SiO_2_-115**). The discolored powder was subsequently separated from the acidic solution by centrifugation (15,000 rpm), followed by four rinsing cycles with bi-distilled water. After resuspending the powder in water, the pH was checked and eventually brought to 6–7 with 0.1 M NaOH micro additions. The solid was further washed with water via centrifugation three times and dried in an oven at 70 °C for 16 h.

#### 3.3.9. Reuptake of Ag-ufNP (8 nm) by **void@SiO_2_-8**, **void@SiO_2_-18**, **void@SiO_2_-115** and SiO_2_ Powders

A total of 160 mg of the chosen grinded powder was suspended in a vial containing 10 mL of citrate-coated Ag-ufNP (8 nm) at the chosen Ag concentration (5.8 × 10^−5^–5.8 × 10^−4^ M range, or 1 × 10^−2^ M) and gently magnetically stirred at room temperature for the chosen time (1–72 h range). The solid powder was then removed via centrifugation (2 cycles: first 5 min at 13,000 rpm, second 5 min 15,000 rpm). Under these conditions, only the grinded silica particles and the reuptaken AgNPs adhering to them are collected in the pellet, while free Ag-ufNP remain in the supernatant. Their concentration was determined via absorption spectroscopy, measuring the absorbance on the maximum of the LSPR band (Abs_reuptaken_). For a correct comparison, a solution of citrate-coated Ag-ufNP (8 nm) at the same concentration and with no added silica powder underwent the same cycles of centrifugation, and its absorbance on the LSPR maximum was measured (Abs_AgNP_). The relative % uptake of Ag-ufNP was determined as follows:

% uptaken Ag = 100 × (Abs_AgNP_ − Abs_reuptaken_)/Abs_AgNP_

It has to be noticed that this type of procedure was not successful when larger NPs were used. As a matter of fact, under the same centrifugation conditions (13,000 rpm), most of the Au-ufNP (18 nm) and AuNP (115 nm) fell down to form a pellet at the bottom of the tube. This did not allow us to discriminate between free and reuptaken NPs.

## 4. Conclusions

An efficient method has been found to prepare silica materials with embedded spherical gold and silver nanoparticles with different diameters. Fast and efficient chemical routes for the chemical oxidation of the embedded nanoparticles were also found, and we obtained nanoparticle-imprinted silica gel, with spherical cavities of the same shape and size as the removed particles. We have shown that the nanoparticle-imprinted silica gels are capable of uptaking uf-AgNP of 8 nm diameter from water, with high efficiency (up to 6.75 μg Ag/mg SiO_2_), although with a relatively slow kinetic, with the process being complete in ~72 h. Moreover, a remarkable size selectivity has been found in the uptake process, with the affinity for 8 nm Ag-ufNP scaling with the cavity dimensions as 8 nm >> 18 nm >> 115 nm >> infinite radius of the cavity (i.e., not imprinted silica). While the use of nanoparticle-imprinted polymeric materials as sensors for metal nanoparticles has been reported [24,25,26], to the best of our knowledge, this is the first report on materials whose intended use is the massive removal of silver nanoparticles from water. Although this paper is still at a proof-of-concept level, the use of nanoparticle-imprinted silica can be imagined on a large scale, e.g., to recover nanoparticles from polluted waste waters. In the case of AgNP, we have also shown that inexpensive green methods (Fe^3+^, O_2_/cysteamine) are available to quickly transform nano silver in Ag^+^ and remove it from silica, with this allowing the recovery of such an expensive element. Finally, on the basis of the observed dimensional selectivity we have indicated that the silica-Ag-ufNP interaction is due to van der Waals attractive forces, optimized for a good cavity–particle size match. As van der Waals interactions are active for any material, and considering that the cavities size can be regulated by choosing the appropriate imprinting Ag or AuNP, the nanoparticle-imprinted silica gels described in this paper could the first step towards devices capable of removing a large-spectrum of nanomaterials from the environment.

## Figures and Tables

**Table 1:** Porosimetry data.

	B.E.T. Specific Surface Area (SSA) (m^2^/g)	SSA Contributed by Micropores (%)	Average Pore Radius Calculated at P/P0 = 0.99 (nm)	Pore Radius by BJH (nm)
**Ag@SiO_2_-8** (monolith)	609.9 (26)	2.08 (0.22)	1.49 (0.05)	1.71 (0.02)
**void@SiO_2_-8** (monolith)	657.2 (29)	20.07 (0.81)	1.35 (0.02)	1.62 (0.08)
**Ag@SiO_2_-8** (powder)	492.7	43.77	1.17	1.53
**void@SiO_2_-8** (powder)	669.4	46.12	1.16	1.53
**Au@SiO_2_-115** (monolith)	600.5	72.85	0.97	1.53
**void@SiO_2_-115** (monolith)	663.4	74.03	0.98	1.53
SiO_2_ (monolith)	724.9 (27)	54.1 (11)	1.21 (0.11)	1.65 (0.85)

**Table 2:** % uptake of 8 nm Ag-ufNP by **void@SiO_2_-8** on variation of the colloidal solution concentration.

Total Ag (M)	% Uptaken Ag	Uptaken Mass (μg)	Uptaken NP Number	% Occupied Cavities
5.8 × 10^−5^	86%	53.7	1.91 × 10^13^	5.3
1.16 × 10^−4^	73%	91.5	3.25 × 10^13^	9.1
2.9 × 10^−4^	65%	203.9	7.25 × 10^13^	20.2
5.8 × 10^−4^	60%	591.1	2.10 × 10^14^	58.7

## Data Availability

The data presented in this study are available in the article text and in the Supplementary Material.

## Supplementary Materials

The following supporting information can be downloaded at: https://www.mdpi.com/article/10.3390/molecules28104026/s1, Figure S1: TGA (thermogravimetric analysis) on Ag-ufNP (8 nm) coated with HS-PEG_750_; Figure S2: absorption spectra on the stability of sols containing Ag-ufNP: Figure S3: Photos of silica gel monoliths containing Ag-ufNP at different total Ag concentrations; Figure S4: absorption spectra on **Ag@SiO_2_-8** monoliths dipped in water or in oxidizing solutions (0.5 M cysteine/air; 0.5 M Fe^3+^); Figure S5: XRD on **Au@SiO_2_-115** and **void@SiO_2_-115** powders from grinded monoliths; Figure S6: porosimetry; Figure S7: geometrical model for NP-imprinted SiO_2_ interaction.
