# Supplementary material for: Nanoparticle-Imprinted Silica Gel for the Size-Selective Capture of Silver Ultrafine Nanoparticles from Water
Source: Molecules. 2023 May 11;28(10):4026. doi: 10.3390/molecules28104026 (PMC10223345; doi:10.3390/molecules28104026)
Supplement: Supplementary file 1 [file molecules-28-04026-s001.zip › molecules-2358300-supplementary.pdf]

Piersandro Pallavicini, Luca Preti, Maria L. Protopapa, Daniela Carbone, Laura Capodieci, Yuri A. Diaz Fernandez, Chiara Milanese, Angelo Taglietti, and Lavinia Doveri

**Nanoparticle-imprinted silica gel for the size-selective capture of silver ultrafine nanoparticles from water**

**Figure S1**. TGA (thermogravimetric analysis) on Ag-ufNP (8 nm) coated with HS-$PEG_{750}$.

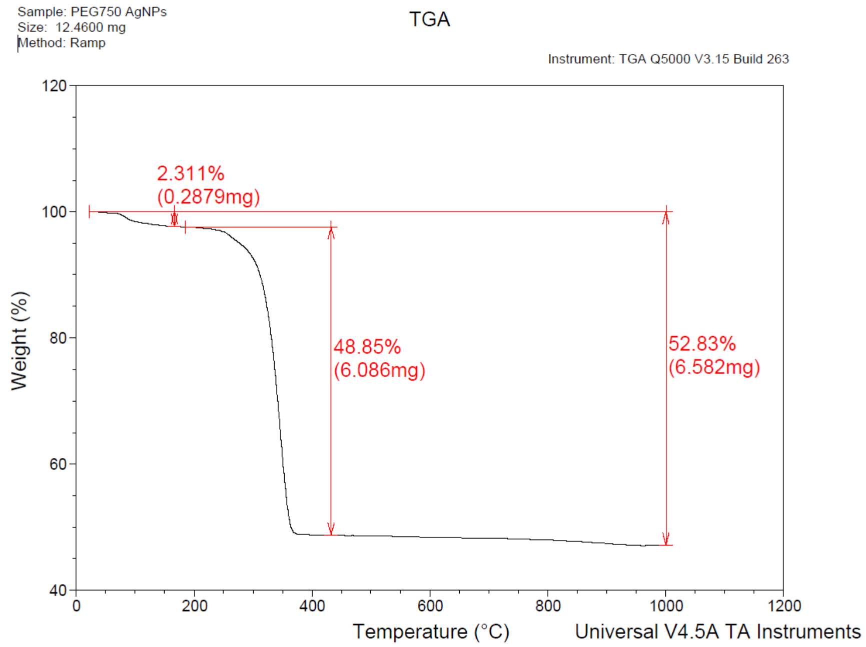


Mass loss at T < 200 °C is adsorbed water; mass loss at 320 °C is HS-PEG (mw 750). The remaining mass % (100-2.311-48.85 = 47.17). From this data, from Ag atomic weight and density, and considering spherical Ag particles of 8 nm (as determined by TEM), the average number of HS-$PEG_{750}$ molecules per nanoparticle is calculated.

**Figure S2.** Stability of sol-gel mixtures.

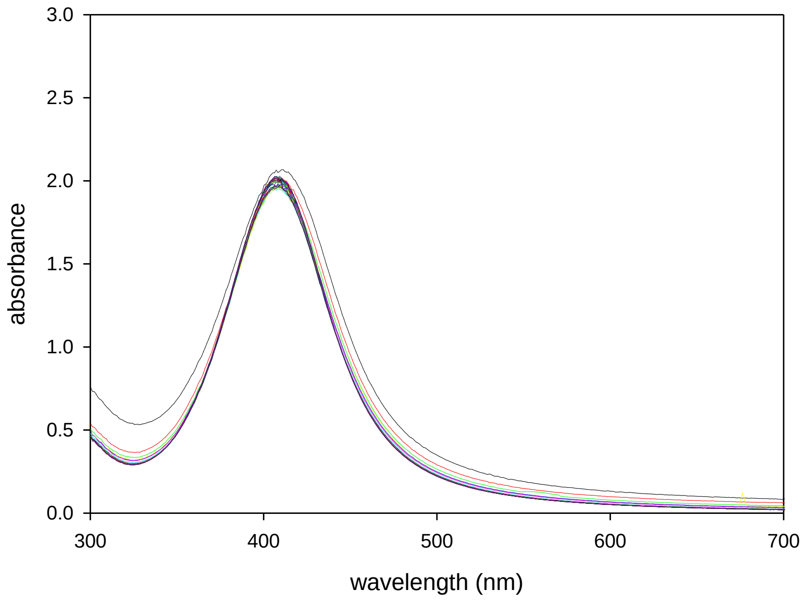


Absorption spectra of a gelating solution in basic conditions (DMF included), containing Ag-ufNP (8 nm; Ag $5.8 \times 10^{-4}$ M, spectra recorded at 10 min intervals for 3 hours

**Figure S3**. Photos of silica gel monoliths containing Ag-ufNP at different total Ag concentrations in the starting solution.

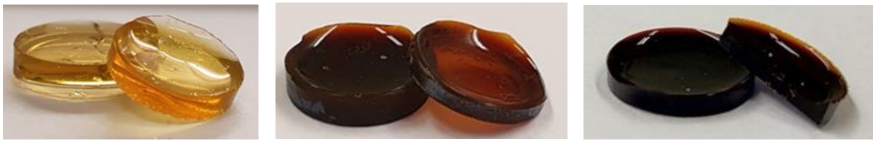

| | | | |
|---|---|---|---|
| Ag tot. (mol/L) | $5.8*10^{-5}$ | $4.66*10^{-4}$ | $9.3*10^{-3}$ |

**Figure S4.** oxidation of **Ag@SiO2-8** monoliths.

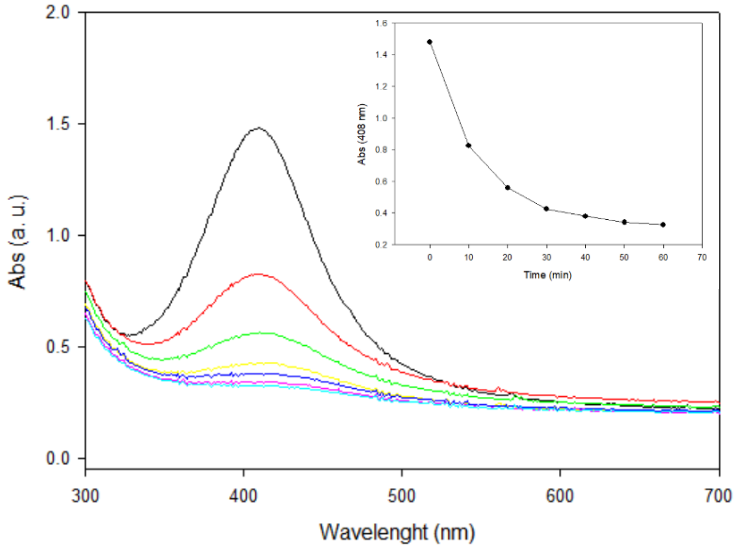


A: absorption spectra on a **Ag@SiO2-8** monolith dipped in 0.5 M cysteine, exposed to air (spectra recorded at 10 min intervals). Inset: Abs on absorption maximum vs time

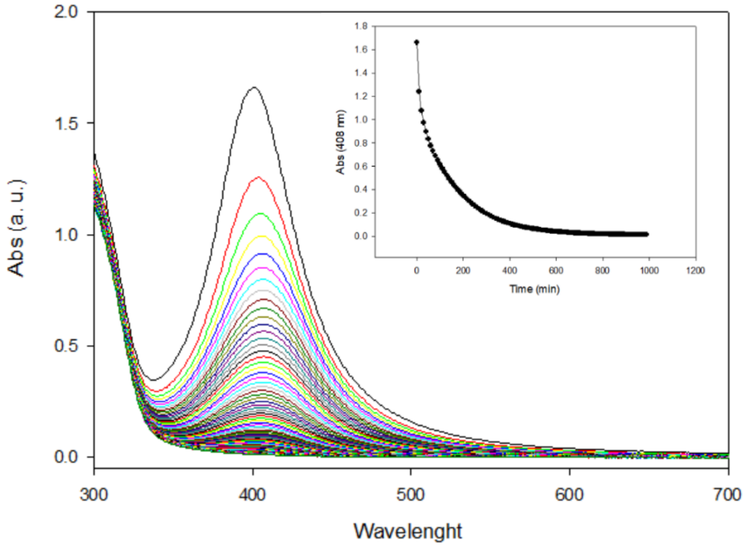


B: same, in 0.5 M Fe(NO3) 3

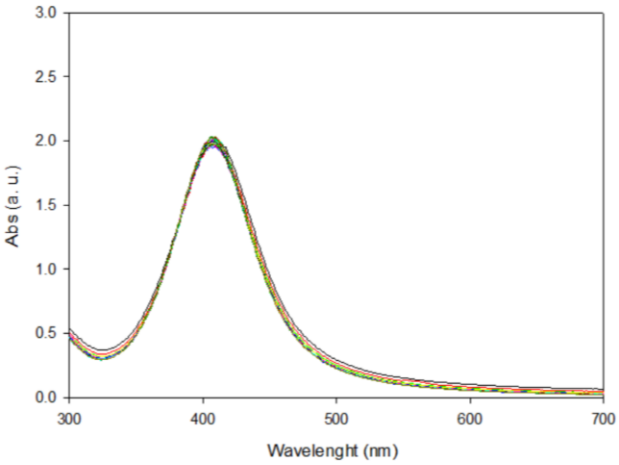


**C**: control (spectra on a **Ag@SiO2-8** monolith dipped in bidistilled water)

**Figure S5.** XRD on **Au@SiO$_2$-115** and **void@SiO$_2$-115** powders from grinded monoliths.

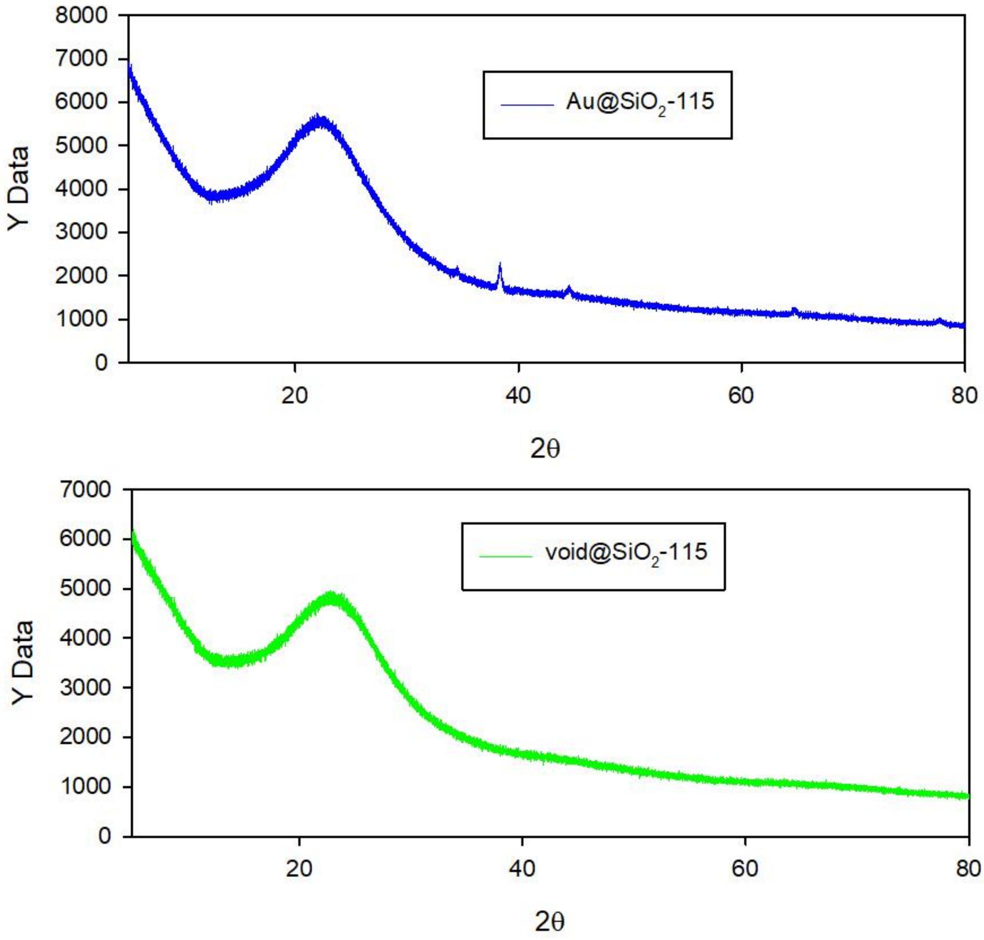

**Figure S6.** Surface area analysis.

**Quantachrome® ASiQwin™- Automated Gas Sorption Data Acquisition and Reduction**
**© 1994-2012, Quantachrome Instruments**
**version 3.0**

Quantachrome INSTRUMENTS

| **Analysis** | | | | | |
|---|---|---|---|---|---|
| **Operator:** | User | **Date:**2020/07/21 | **Report Operator:** | daniela | **Date:**2020/10/08 |
| **Sample ID:** | sample A | **Filename:** | sample A.qps | | |
| **Sample Desc:** | sample A | **Comment:** | cella 12mm senza bulbo | | |
| **Sample Weight:** | 0.0565 g | **Instrument:** | Autosorb iQ Station 1 | | |
| **Approx. Outgas Time:** | 3.1 hrs | **Final Outgas Temp.:** | 120 °C | **Extended info:** | Available |
| **Analysis gas:** | Nitrogen | **Non-ideality:** | 6.58e-05 1/Torr | **CellType:** | 12mm |
| **Analysis Time:** | 2:34 hr:min | **Bath temp.:** | 77.35 K | | |
| **Analysis Mode:** | Standard | | | **VoidVol Remeasure:** | off |
| **VoidVol. Mode:** | He Measure | **Cold Zone V:** | 2.36307 cc | **Warm Zone V:** | 8.93303 cc |

## Isotherm : Linear

**Data Reduction Parameters**

| | | | |
|---|---|---|---|
| **Adsorbate** | **Thermal Transpiration:** on | **Eff. mol. diameter (D):** 3.54 Å | **Eff. cell stem diam. (d):** 4.0000 mm |
| | Nitrogen | **Temperature** 77.350K | |
| | **Molec. Wt.:** 28.013 | **Cross Section:** 16.200 Å² | **Liquid Density:** 0.808 g/cc |

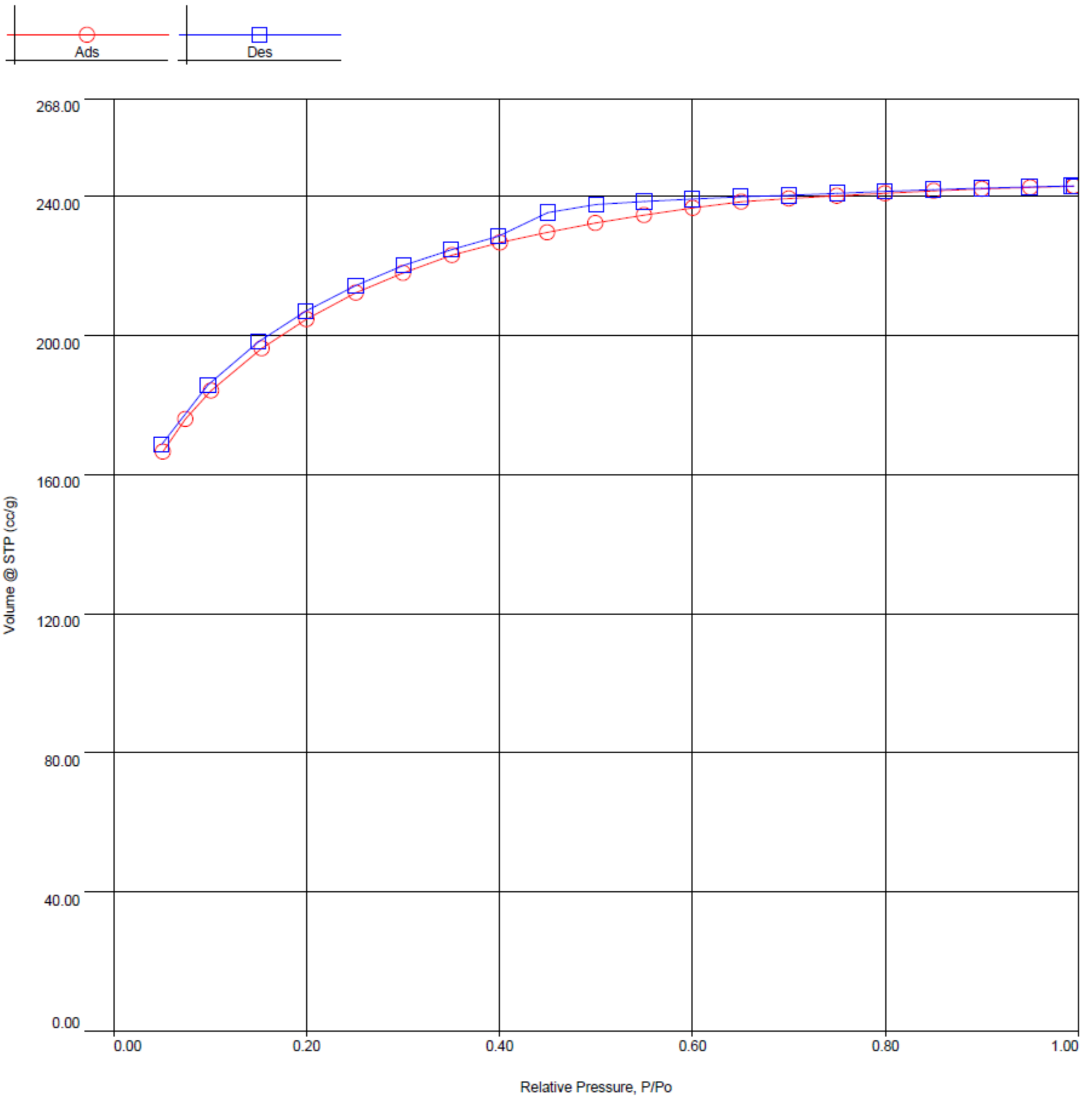


Quantachrome® ASiQwin™- Automated Gas Sorption Data Acquisition and Reduction © 1994-2012, Quantachrome Instruments version 3.0

Report id:{1110142135:20201008 184605639} Page 1 of 1

**A** – Adsorption and desorption isoterms for **Ag@SiO$_2$-8**

**Quantachrome® ASiQwin™- Automated Gas Sorption Data Acquisition and Reduction**
**© 1994-2012, Quantachrome Instruments**
**version 3.0**

| **Analysis** | | | **Report** | | |
|---|---|---|---|---|---|
| **Operator:** | User | **Date:**2020/07/16 | **Operator:** | daniela | **Date:**2020/10/19 |
| **Sample ID:** | sample B1 | **Filename:** | sample B1.qps | | |
| **Sample Desc:** | silice+ AgNps+cisteamina | **Comment:** | cella 12mm senza bulbo | | |
| **Sample Weight:** | 0.0561 g | **Instrument:** | Autosorb iQ Station 1 | | |
| **Approx. Outgas Time:** | 3.1 hrs | **Final Outgas Temp.:** | 120 °C | **Extended info:** | Available |
| **Analysis gas:** | Nitrogen | **Non-ideality:** | 6.58e-05 1/Torr | **CellType:** | 12mm |
| **Analysis Time:** | 2:36 hr:min | **Bath temp.:** | 77.35 K | | |
| **Analysis Mode:** | Standard | | | **VoidVol Remeasure:** | off |
| **VoidVol. Mode:** | He Measure | **Cold Zone V:** | 2.18258 cc | **Warm Zone V:** | 9.09856 cc |

## Isotherm : Linear

**Data Reduction Parameters**

| **Adsorbate** | **Thermal Transpiration:** on | **Eff. mol. diameter (D):** 3.54 Å | **Eff. cell stem diam. (d):** 4.0000 mm |
|---|---|---|---|
| | Nitrogen | **Temperature** 77.350K | |
| | **Molec. Wt.:** 28.013 | **Cross Section:** 16.200 Å² | **Liquid Density:** 0.808 g/cc |

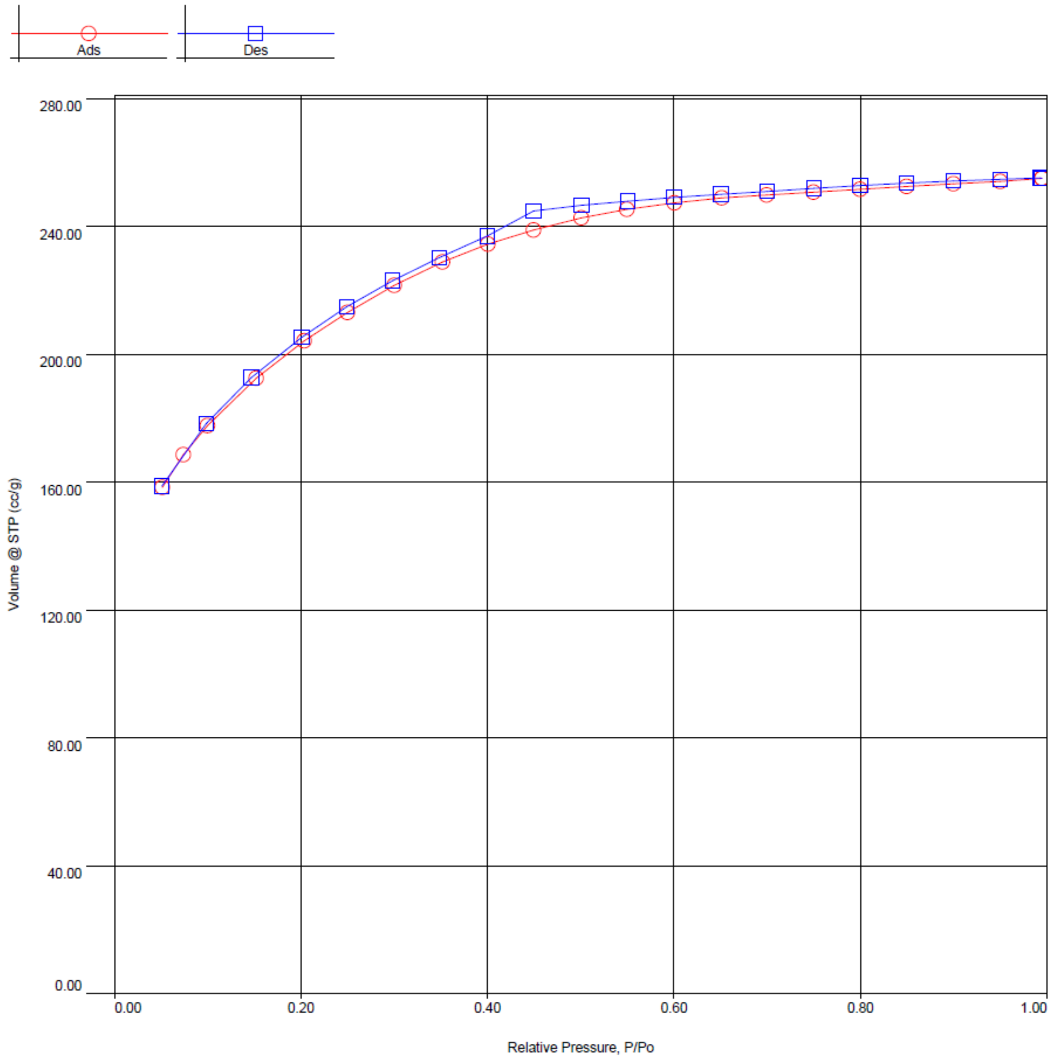


Quantachrome® ASiQwin™- Automated Gas Sorption Data Acquisition and Reduction © 1994-2012, Quantachrome Instruments version 3.0

Report id:{485289492:20201019 141726789} Page 1 of 1

**B** – Adsorption and desorption isoterms for **void@SiO$_2$-8**

**Figure S7.** Geometrical model for size selectivity.

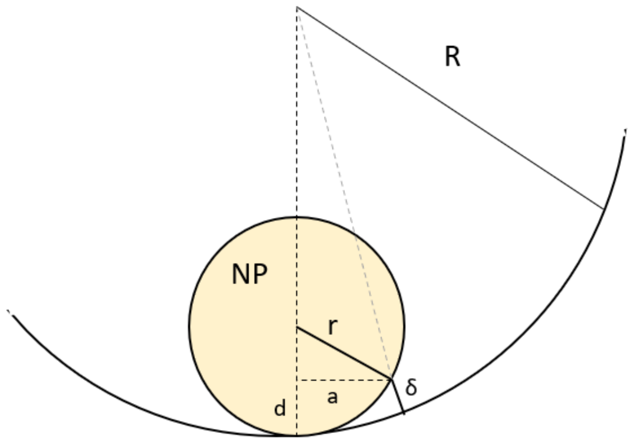


**A**: geometrical features for a spherical NP (r radius) interacting with a spherical cavity (R radius of curvature)

When a spherical NP interacts with a curved surface, such as the inner surface of a cavity, it is possible to calculate the fraction of the particle surface interacting with the curved cavity. In this case, the fraction of interacting NP surface will depend on the ratio between the particle size (r) and the radius of curvature of the cavity (R), leading to a relative increase of the particle-cavity interactions as this ratio increases (see Figure SM7B). In fact, within a curved cavity, we can expect a larger fraction of the NP surface to be close enough to the solid material, compared to a flat surface of the same material. This effect can be modelled using simple geometrical considerations (Figure SM7A), leading to the conclusion that the interactions will be stronger when the particle size and the radius of curvature of the cavity are similar.

Triangle 1: $(R-\delta)^2 = a^2 + (R-d)^2$

riangle 2: $r^2 = a^2 + (r-d)^2$

$(R-\delta)^2 - r^2 = (R-d)^2 - (r-d)^2$

$(R-\delta)^2 - r^2 = (R+r-2d)(R-r)$

$$d = -\frac{(R-\delta)^2 - r^2}{2(R-r)} + \frac{1}{2}(R+r)$$ With R≠r

$$d = \frac{1}{2}[(R+r) - \frac{(R-\delta)^2 - r^2}{(R-r)}$$

This simple geometrical model can explain the selectivity of the silica materials decorated with cavities, in the reuptake process of AgNPs.

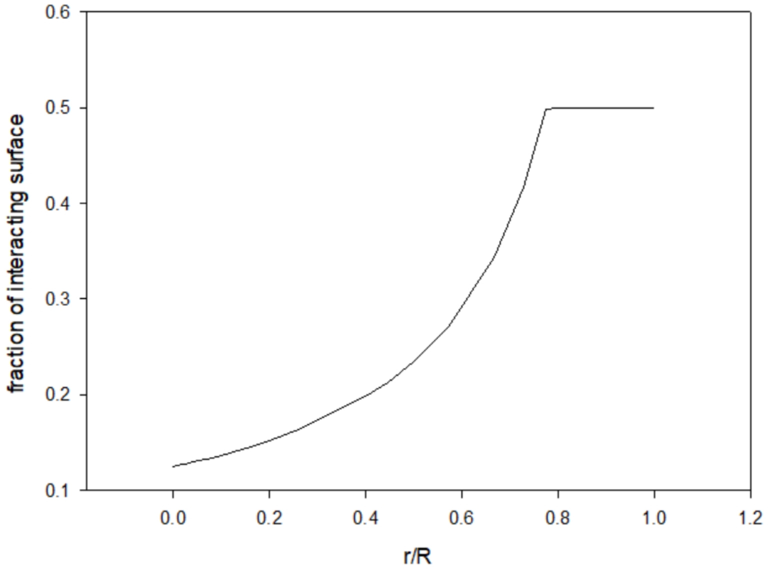


**B**: increasing of interacting surface as the reaching of the R value.

It is important to note that within this model we have assumed that cavities are half-hemispheres, to allow for the NP to enter (i.e. when the size of the NP is close to the cavity radius, the NP can only enter the cavities that have been cut through at least by half). For this reason, the limiting value of the NP surface fraction is 0.5 when NP and cavity sizes become equal. This assumption is not limiting the overall interpretation of the results and become irrelevant for cavities much larger than the NP size.

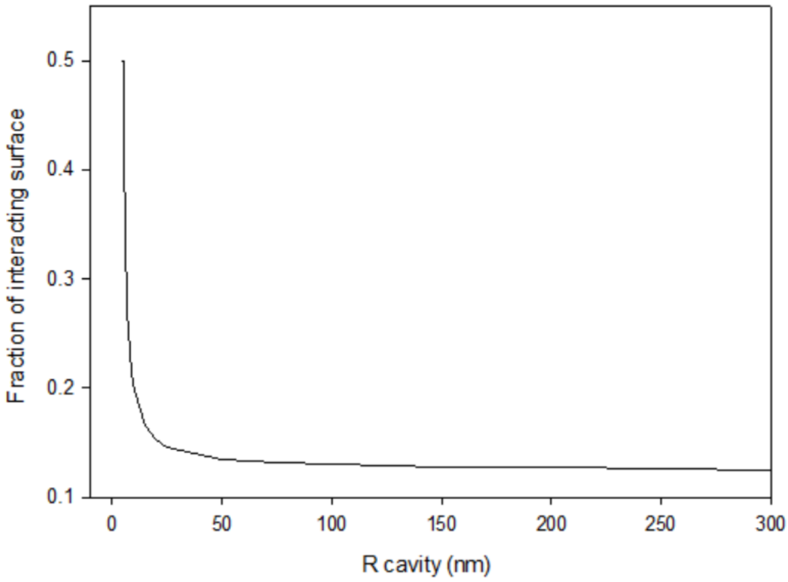


**C**: fraction of interacting surface VS the cavity size.
